# Depression, suicidality and antidepressants: A coincidence?

**DOI:** 10.4103/0019-5545.58890

**Published:** 2010

**Authors:** Vithyalakshmi Selvaraj, Snehamala Veeravalli, Sriram Ramaswamy, Richard Balon, Vikram K. Yeragani

**Affiliations:** Department of Psychiatry, Creighton University School of Medicine, Omaha, Nebraska, India; 1 Department of Psychiatry, Jamaica Hospital Medical Center, Jamaica, New York; 2 Department of Psychiatry and Behavioral Neurosciences, Wayne State University School of Medicine, Detroit, MI, U.S.A; 3 Department of Psychiatry, University of Alberta, Edmonton, Alberta, Canada, India; 4 Department of Biotechnology, Aacharya Nagarjuna University, A.P., India

Major depressive disorder is currently one of the leading causes of disability world-wide and projected by the World Health Organization (WHO) to become the second leading cause of disability after heart disease by the year 2020. Suicidal ideation and behavior are the most serious and common psychiatric emergencies among the depressed population. In 2004, suicide was the eleventh leading cause of death in the U.S.[1] The overall rate was 10.9 suicide deaths per 100,000 people. An estimated 8 to 25 attempted suicides occur per every suicide death. It is reassuring that depressed patients usually respond to treatment with antidepressants. In fact, population-based studies examining rates of antidepressant use and suicide over time, have generally found lower rates of suicide with increasing rates of antidepressant use.[2–8] However, in 2003, publication of data linking antidepressant use to suicide and suicidal behavior in children and teenagers sparked off worldwide public health concern. This prompted the US Food and Drug Administration (FDA) to mandate manufacturers of all antidepressants (including tricyclic antidepressants [TCAs] and monoamine oxidase inhibitors) to include a warning stating that antidepressants may increase the risk of suicidal ideation and behavior in children, adolescents and young adults less than 25 years old. The ‘black box’ warning, although meant to safeguard public health has had serious spillover effects on pharmacotherapy of depression. There have been recent reports of a rise in suicide rates among the youth and a decline in depression care among the older adults. We critically evaluate this association in order to provide more clarity while assessing the benefits of antidepressant use versus any risk of self-harm associated with them.

## IMPACT OF DEPRESSION

Depression is a pathological and pervasive state of mood. The lifetime risk of developing major depressive disorder is 16.2% in the United States. Recurrence is the rule, and about one-third of patients go on to develop chronic depression. Depression increases mortality because it worsens many medical conditions such as cardiovascular disease and diabetes and increases the risk of suicide. Depressed patients are high utilizers of healthcare services and typically perform poorly in the workplace. The mean age of onset, from a number of studies, is in the late 20s. Although twice as many females as males report or receive treatment for clinical depression, this gap has significantly narrowed in the recent past. Also, this difference disappears after the age of 50-55, when most females are post-menopausal. Suicidal ideation and attempts are common in depressed patients. Each year, about 30,000 people in the United States and one million worldwide die by suicide. Approximately 650,000 people in the United States receive emergency treatment each year after attempting suicide.

## ANTIDEPRESSANTS BENEFITS AND RISKS

Mono amine oxidase inhibitors (MAOI) and tricyclic antidepressants (TCA) were the mainstays of treatment for depression until the introduction of Serotonin Reuptake Inhibitors (SSRIs) in the late 1980's. Currently, SSRIs are the most commonly prescribed antidepressants. The therapeutic effects of antidepressants are believed to be related to their ability to facilitate serotonin, norepinephrine and/or dopamine neurotransmission. Despite the early increase in concentration of monoamine neurotransmitters at receptor sites, clinical antidepressant benefits are not evident until several weeks. One explanation for this therapeutic lag is the theory that “down-regulation” of neurotransmitter receptors is a consequence of excess signaling, a process that takes several weeks and ultimately responsible for the alleviation of depressive symptoms. Overall, antidepressants are very effective in treating depression and its associated complications. However, there is substantial disagreement and controversy about whether antidepressants trigger suicidal ideation and if they do, whether this risk outweighs the therapeutic benefits of these medications. Several confounding factors such as severity of depression, rates of alcohol and drug abuse, undiagnosed bipolar illness, impulsivity and treatment compliance must be considered in order to prove causality. Also, many of the early reports of suicide risk with SSRIs came from randomized clinical trials, which were underpowered to detect a relationship between SSRI use and suicidal ideation or behavior.

## SSRI AND SUICIDALITY

While there were occasional reports of antidepressant induced suicidality in the 1970s and 1980s, the concerns intensified in 1990 with an article published in the American Journal of Psychiatry. Teicher[9] and co-investigators at Harvard Medical School reported on six depressed patients, who in their view had become suicidal as a result of treatment with the SSRI, fluoxetine. Numerous studies have attempted to find associations between SSRIs and suicide by examining population-based rates of antidepressant use and suicide-risk over time. Both population-based studies and observational studies have not consistently shown an increased risk in patients treated with SSRIs compared with TCAs. Suicide is rare in randomized, controlled trials (RCTs) of antidepressants. For this reason, individual trials typically lack the power to prove or exclude the possibility that antidepressants cause suicidal ideation or behavior. Large meta-analyses and systematic reviews have looked for evidence that antidepressants may increase the risk of suicide, suicidal ideation, or self harm. However, even these analyses have only limited power to detect an effect on the very rare outcome of suicide.[10–12] After review of the literature, we chose to mention the results of three studies based on clinical trials.

Khan *et al*,[13] analyzed FDA summary reports of the controlled clinical trials for 9 antidepressants (fluoxetine, sertraline, paroxetine, nefazodone, mirtazapine, bupropion, venlafaxine both immediate and extended release and citalopram). They tested for differences in reported rates of suicide among depressed patients randomly assigned to treatment with an investigational (FDA –approved) SSRI antidepressant compared with similar subjects assigned to another standard antidepressant or to placebo. Rates of suicides were classified for patients assigned to SSRI, placebo or other class of antidepressants. The SSRIs were fluoxetine, sertraline, paroxetine, citalopram, and among the SSRI group they had an active control, fluvoxamine. The other antidepressants were the investigational agents, nefazodone, mirtazapine, bupropion, and venlafaxine as well as six active controls, imipramine, amitriptyline, maprotiline, trazodone, mianserin, and dothiepin.

There were 48, 277 patients that were followed-up. Out of this sample, 26,109 patients were assigned to SSRIs, 17273 were assigned to other antidepressants, and 4,895 were assigned to placebo. For purposes of data analysis, the authors scrutinized all the suicides from all FDA summary bases of approval reports and listed the antidepressant the patients were taking at the time of suicide. They attempted to assess both rates of suicide and suicide attempts. They included only completed suicides for this report because it was hard to classify by specific agent that may have caused the suicide attempt. They used chi-square analyses to assess the statistical significance of differences in suicide frequency among the subjects receiving placebo, SSRIs, and other antidepressant medication.

There was no statistically significant difference in crude suicide rates among patients assigned to SSRIs, other antidepressants, or placebo (*P*>0.05). When groups were compared on the basis of patient-exposure years, there was no statistically significant difference in suicide rate among patients assigned to SSRIs, other antidepressants or placebo. Of the 48,277 depressed patients participating in the trials, 77 committed suicide. Out of 26,109 patients assigned to SSRIs there were 38 suicides and 34 out of 17,273 patients on other antidepressants. Placebo was associated with five suicides among patients out of 4,895.

In the second study, a controlled forensic database of 14,857 suicides was examined by Isacsson, and co-investigators between the years 1992-2000 in Sweden.[14] This study was aimed at evaluating the suicidal outcome of the total nationwide use of antidepressants during the nine years by analysis of all the forensic toxicological investigations of suicides. Here the hypothesis was that if SSRI treatment increases the suicide risk in depressed individuals, the incidence of suicide should be observed more often than otherwise expected.

In this study, data from the department of Forensic Chemistry of the National Board of Forensic Medicine in Sweden, which conducts forensic investigation on unnatural deaths were included. About 200 substances were screened for, including all antidepressants in femoral blood.[15,16] During these years 14,857 suicides (median age 49 years, 71% men) including 4,301 uncertain cases in regards to death were investigated.

A control group was included in order to provide a control for the varying prescription rates of different antidepressants in general population, including their varying probability of being detected in toxicological screening at low concentrations. The control group consisted of 26,422 patients which after the forensic investigation, were judged to be natural deaths or accidents (median age was 55 years and 73% were men). The rates at which different antidepressants were found, in controls as well as in subjects that committed suicides, may, however, reflect their respective sales in the general population. To validate this, additional data regarding antidepressants sales on prescription to different age groups were collected from National Corporation of Pharmacies as the number of defined daily doses. Due to the low number of suicides in children and adolescents, relative risks with 95% CI were also calculated in the suicide group, with the sales data as the measurement for exposure.

The total use of antidepressants in the age group under 15 years of age calculated from the sales data was 2817 person-years. Among 52 cases of suicide, seven were positive for clomipramine, imipramine, mianserine and venlafaxine and no SSRIs were detected. Among 998 in the control group, four were positive for antidepressants, one for amitriptyline, and three for citalopram.

In the 15-19 year age group, the use of antidepressants amounted to 14,128 person years. Among 326 cases of suicide in this age group, there were 13 cases, which were positive for antidepressants. Among 577 controls, five were positive for anti depressants (amitriptyline, clomipramine, citalopram, fluoxetine and sertraline). The relative risks for SSRIs were lower (0.14) when compared with that of non-SSRIs.

The third study by Yerevanian[17] and colleagues examined the rates of suicidal behavior for patients maintained on antidepressants versus rates of suicidal behavior after discontinuation of the medication. All patients were carefully followed-up by a single clinician throughout the period of study, and medication compliance was documented. This study comprises of carefully followed clinical population of patients with unipolar depression in order to better clarify the details of antidepressant effects on suicide. This study is a retrospective review of the clinical records of 521 patients followed for various durations of treatment by the senior author between 1978 and 2002, in the U.S.A., spanning the era of TCAs as well as the SSRIs after their introduction in 1987. Dependent variables were completed suicide, suicide attempts, defined as self-induced physical damage with expressed intent to die and, hospitalization for suicidal thought or intent. Dependent variables in the report were only those that happened during time under the author's care. A hierarchical system was constructed such that for any episode, the more serious event was reported. The measure of independent variables was the time recorded as total time observed rather than time to event. For many patients this includes both time before and after any suicide event. Normal curve ‘Z’ tests and two tailed probabilities were computed by dividing the rates by their standard errors.

Rates of all suicidal events increased more than five-fold during periods after discontinuation of antidepressants, seven-fold increases in the suicide attempts and fourfold increase in hospitalization in OFF periods. Effects of ON versus OFF periods for TCAs were more than five-fold during the OFF-TCA period for both hospitalization and suicide attempts compared with the ON period. All suicide events were more than four-fold higher during the off period for the SSRIs. The rates for all suicide events considered together were significantly higher during treatment periods with TCAs than they were during treatment with the SSRIs (*P*=0.046). When each category of suicide compared individually there were no significant differences.

A few other studies also need to be mentioned in this context. For example, in a pooled analysis of suicidality in double-blind, placebo controlled studies of sertraline in adults, Vanderburg and colleagues[18] did not find significant increase in suicidality risk in adult sertraline versus placebo-treated patients. Similarly, in a large meta-analysis by British authors,[12] there was no evidence that SSRIs increased the risk of suicide. However, Baldessarini and colleagues[19] in their analysis of 19 ecological studies of antidepressant treatment and suicide concluded that the findings of these studies yielded limited and inconsistent support for the hypothesis that increased use of modern antidepressants might limit suicide risk. They also concluded that there was no evidence from these studies to show that the suicidal risk increased. As one of us[20] noted several years ago, the evidence for the answer to the question whether SSRIs specifically trigger suicidality (vague term!) seemed to be in the eye of the beholder. That still seems to be the case, even more.

As Baldessarini and colleagues[19] pointed out, suicide risk is determined by complex factors. Rather than focusing solely on the presumable but unconfirmed risk of suicide associated with SSRIs, clinicians should focus on the overall assessment of suicide risk. For example, a recent naturalistic study by Seemuller[21] and colleagues determined five risk factors for emergence of suicidal ideation in hospitalized patients: age over 45 years, treatment resistance, number of hospitalizations, presence of akathisia and co morbid personality disorder.

Current and future studies may be inadequate to conclusively prove or disprove an association between the SSRIs and suicidal ideation or behavior in adults. Nevertheless, it appears that the SSRIs are quite safe in this regard and the benefits outweigh any risks by a large extent. Any increase in the risk of non-fatal self harm appears to be very small. A small proportion of patients treated with SSRIs may become restless and others may show increases in anxiety in the initial phase of treatment, but no increased susceptibility to aggression or suicidality can be connected with fluoxetine or any other SSRIs. On the contrary, SSRI treatment may reduce aggression, probably due to their effects on the serotonergic dysfunction that is implicated in aggressive behavior directed towards self or others. At the present, we lack concrete evidence indicating that SSRIs and other newer antidepressants increase the risk of suicidal ideation or completed suicide in adults. The SSRIs and other newer antidepressants are efficacious in the treatment of depression in adults. It is recommended to use these medications in the treatment of depressed adults since untreated depression is highly correlated with suicide. Hence, one should not restrict the use of SSRIs when indicated but should be cautious and needs to follow these patients closely, like with any other medication. The ideal way is to conduct randomized trials to examine this hypothesis. But ethical and humanitarian issues limit this kind of studies. Future studies may address the issue in a different light so that premature conclusions can be avoided about any medication that is potentially useful. In the absence of convincing evidence to link SSRIs causally to suicide, the lay media reports are potentially dangerous, unnecessarily increasing the concerns of depressed patients who have been prescribed antidepressants. It is rather wise not to dismiss useful less toxic medications from the market unless there is substantial evidence that they are harmful or dangerous in any way.
